# Low-dose administration of prednisone has a good effect on the treatment of prolonged hematologic toxicity post-CD19 CAR-T cell therapy

**DOI:** 10.3389/fimmu.2023.1139559

**Published:** 2023-03-14

**Authors:** Jiaxi Wang, Meng Zhang, Hairong Lyu, Ruiting Guo, Xia Xiao, Xue Bai, Yedi Pu, Juanxia Meng, Qing Li, Ting Yuan, Wenyi Lu, Mingfeng Zhao

**Affiliations:** Tianjin First Central Hospital, The First Central Clinical College of Tianjin Medical University, Tianjin, China

**Keywords:** CAR-T cells, acute lymphocytic leukemia, prolonged hematologic toxicity, prednisone, hematopoietic recovery

## Abstract

**Introduction:**

Hematologic toxicity (HT) is a joint adverse event after CAR-T cells infusion. Some patients experience prolonged hematologic toxicity (PHT), which is challenging to treat.

**Methods:**

We collected clinical data from patients with relapsed refractory B-ALL treated with CD19 CAR-T cells. Patients with PHT who did not respond to erythropoietin, platelet receptor agonists, transfusion, or G-CSF and eventually received low-dose prednisone therapy were included in the analysis. We retrospectively analyzed the efficacy and safety of low-dose prednisone on PHT.

**Results:**

Among 109 patients treated with CD19 CAR-T cells, 78.9% (86/109) of patients were evaluated as PHT. Of these, 15 patients had persistent hematological toxicity after infusion (12 were grade 3/4 cytopenia, 12 were trilineage cytopenia and 3 were bilineage cytopenia), 2 developed cytopenia without apparent cause after D28. The initial prednisone dose was 0.5 mg/kg/day, and the median response time was 21 days (7-40 days). The recovery rate of blood count was 100%, and the complete recovery rate ranged from 60% to 66.67%. Especially exciting was that HT recurred in 6 patients after stopping prednisone. They were relieved again after the administration of prednisone. The median follow-up time was 14.97 months (4.1-31.2 months). Twelve-month duration of PFS and OS rates were 58.8% (±11.9%) and 64.7% (±11.6%). We did not observe any other side effects of prednisone apart from drug-controllable hyperglycemia and hypertension.

**Discussion:**

We suggest that low-dose prednisone is a beneficial and tolerable therapy for PHT after CAR-T cells. The trials have been registered at www.chictr.org.cn as ChiCTR-ONN-16009862 (November 14, 2016) and ChiCTR1800015164 (March 11, 2018).

## Introduction

The efficacy of chimeric antigen receptor T (CAR-T) T cells for treating hematologic malignancies has been widely recognized. CD19 CAR-T cells have achieved complete remission (CR) rates of over 90% for B-cell acute lymphocytic leukemia (B-ALL) (1). The usual adverse events after CAR-T cells are cytokine release syndrome (CRS) and immune effector cell-associated neurotoxicity syndrome (ICANS), with an incidence of approximately 81% and 40%, respectively (2). However, little attention has focused on hematologic toxicity (HT), with the incidence as high as 90% (3–7). In general, approximately 63.3% of cytopenia patients had restored normal blood count at 13 months after transfusion (8, 9), and 16% of patients still had hemocytopenia even at the follow-up of 22 months (10). Prolonged hematologic toxicity (PHT) increases the incidence of infection, bleeding, and fatigue in patients, which should be taken seriously.

Higher tumor burden, multiple lines of prior therapy, and pretreatment with lymphodepletion may impair bone marrow hematopoiesis (11, 12), which may contribute to the occurrence of PHT after CAR-T cell therapy. Furthermore, some recent studies showed that subsequent malignancies, such as myelodysplastic syndrome (MDS) and clonal hematopoiesis of indeterminate potential (CHIP) (8, 10, 13), occurred in a proportion of PHT patients. However, these factors do not fully explain the occurrence of PHT. PHT may be related to bone marrow suppression triggered by CAR-T cells, increased inflammatory factors, or the activation of excessive immune responses (14).

Glucocorticoids are commonly used to manage high-grade CRS and ICANS. However, the effect of glucocorticoids on PHT is uncertain. In this study, we enrolled 17 patients with relapsed/refractory B-ALL (R/R B-ALL) who developed PHT after CAR-T cell therapy and evaluated the effect and safety of low-dose prednisone on long-term hematologic recovery.

## Methods

### Patients and data collection

We retrospectively analyzed R/R B-ALL patients treated with CD19 CAR-T cells from September 2019 and September 2022. All enrolled patients participated in a single-center clinical trial of CAR-T cell therapy targeting CD19 (ChiCTR-ONN-16009862 and ChiCTR1800015164). The inclusion criteria were as follows: 1) diagnosis of R/R B-ALL, 2) treatment with CD19 CAR-T cells, 3) CR at D28, 4) HT remained in D28, and 5) receiving low-dose prednisone therapy. The exclusion or termination of follow-up criteria were as follows: 1) bridging to hematopoietic stem cell transplantation (HSCT) or other radiotherapy or chemotherapy regimens after CAR-T cell treatment; 2) secondary to or combining with hematopoietic disorders such as MDS or CHIP; and 3) receiving other treatments or drugs that interfere with the efficacy of prednisone during oral prednisone administration, such as granulocyte colony-stimulating factor (G-CSF), erythropoietin, platelet receptor agonists.

The primary objective was to assess the efficacy and safety of low-dose prednisone in PHT that was ineffective for G-CSF or blood transfusion after CAR-T treatment. The secondary objective was to assess the effect of prednisone on CAR-T cell efficacy.

The study was approved by the institutional review board at Tianjin First Center Hospital and was conducted according to the Good Clinical Practice guidelines of the International Conference on Harmonization. The patients were informed about the treatment regimen’s potential clinical benefits and adverse events (AEs), including CD19 CAR-T cells and glucocorticoids, and provided written informed consent. The Tianjin First Central Hospital Medical Ethics Committee granted ethical approval for this study.

### CD19 CAR-T cell infusion

CD3 T cells were isolated from peripheral blood mononuclear cells of patients or healthy donors by using CD3 immunomagnetic beads and then cultured with a medium containing CD3/CD28 stimulating beads. The lentiviral vector containing CD19-28ζ CAR was then transduced into these cells. Finally, CAR-T cells would expand to a sufficient number for infusion. The transfection efficiency of CD19 CAR-T cells was approximately 50%. Patients would receive cyclophosphamide 300 mg/m^2^ and fludarabine 30 mg/m^2^ daily for 3 to 2 days prior to CAR-T cell infusion, and they were treated with CD19 CAR-T cells on D0. CRS was prospectively graded using the Lee scale (with initial patients retrospectively graded) (15).

### Definition, treatment, and recovery criteria of HT

Patients with anemia, thrombocytopenia, and/or neutropenia on D28 were considered to be experiencing PHT. The criteria for cytopenia were defined according to the Management of Immune-Related Adverse Events in Patients Treated With Chimeric Antigen Receptor T-Cell Therapy: ASCO Guideline (16). The specific standards are shown in **Table 1**.

**Table 1:** The criteria for cytopenia.

Grading	Anemia (g/L)	Thrombocytopenia (×10^9^/L)	Neutropenia (×10^9^/L)
G1	LLN–10.0	>75	>1.5
G2	<10.0–8.0	>50	>1
G3	<8.0	>25	>0.5
G4	Life-threatening	<25	<0.5

G, grade; LLN, lower limit of normal.

Patients who had not responded to G-CSF, blood transfusions, or other similar therapies began to take low-dose prednisone 1 month after CAR-T cell infusion. The initial dose of prednisone was 0.5 mg/kg/day, which was halved after the blood count recovered a grade and gradually reduced until it was discontinued during hematologic recovery. The patients used calcium tablets, gastric mucosa protectors, and anti-fungal drugs to prevent the side effects of prednisone.

We defined hematologic recovery as the absence of blood transfusion and G-CSF support. Recovery was considered when hematocrit recovered to G2, and complete recovery was considered when it returned to standard levels. We defined the median response time as the period from the start of oral prednisolone to the recovery of at least one-degree anemia, thrombocytopenia, or neutropenia.

### Statistical analysis

The results regarding patient characteristics were obtained using descriptive statistics. Overall survival (OS) and progression-free survival time (PFS) were calculated by the Kaplan–Meier analysis. OS was defined as the time from CAR-T cell treatment to death. PFS was defined as the time from CAR-T cell treatment to disease progression or death. Statistical analyses were performed using the SPSS v19.0 software (Chicago, IL, USA) and the GraphPad Prism v9 software (GraphPad, La Jolla, CA, USA).

## Results

### Essential characteristics of patients enrolled

Among 159 patients treated with CD19 CAR-T cells, 109 had evaluated blood count follow-up data, and 78.9% (86/109) of patients were evaluated as experiencing PHT. Fifteen patients did not respond to erythropoietin, platelet receptor agonists, or blood transfusion and G-CSF dependence. Two developed cytopenia without apparent cause after D28. Our analysis included them eventually for treatment with oral low-dose prednisone (**Table 2**); 52.94% (9/17) were male, and the median age was 31 years (range 8–66). The detailed characteristics of the patients are shown in **Table 3**. Except for one patient with the acute lymphoblastic transformation of chronic lymphocytic leukemia, the other patients had B-ALL; 47.06% (8/17) of patients had extramedullary disease, and 29.41% (5/17) had central nervous system infiltration. The median number of therapy lines before CAR-T cell infusion was 5 (range 2–15).

**Table 2:** Patient demographics.

Patient demographics	Total median (n = 109)	PHT group (n = 86)	Non-PHT group (n = 23)
Age, median (range), years	34 (9–68)	33 (11–68)	43 (9–66)
Sex			
Male (%)	57 (53.27)	46 (53.49)	11 (47.83)
Female (100%)	50 (46.73)	40 (46.51)	12 (52.17)
Extramedullary infiltration			
CNS (%)	10 (9.17)	9 (10.47)	1 (4.35)
Others (%)	14 (12.84)	13 (15.12)	1 (4.35)
Previous treatments			
HSCT (%)	60 (56.07)	51 (59.3)	9 (39.13)
Lines of therapy (range)	5 (0–13)	4 (1–12)	5 (0–13)

PHT, prolonged hematologic toxicity; CNS, central nervous system; HSCT, hematopoietic stem cell transplantation.

**Table 3:** Patient and disease characteristics (n = 17).

Patient	Age/sex	Malignancy	Cytogenetics at diagnosis	Extramedullary infiltration	Lines of therapy	Prior HSCT	Blood count			CAR-T cells infused (×10^6^/kg)	Response	CRS	ICANS
							HGB (g/L)	PLT (×10^9^/L)	NE (×10^9^/L)				
1	8/M	B-ALL TELAML1	Untested	Yes	3	No	124	75	1.59	5.1	CR	1	0
2	40/F	B-ALL Ph+	Normal	No	7	No	86	111	0.78	1.5	CR	3	0
3	32/M	B-ALL^*^ JAK2-E890K	Untested	Yes	5	Yes	86	44	0.95	7.65	CR	1	0
4	50/M	B-ALL Ph+	Normal	Yes	2	No	104	370	12.18	3.37	CR	2	0
5	18/F	B-ALL	Normal	No	4	Yes	114	77	5.01	1.5	CR	1	0
6	19/M	B-ALL WT1, NOTCH1	Normal	Yes	4	Yes	126	63	4.76	2.5	CR	1	0
7	34/F	B-ALL	Complex	No	4	Yes	114	77	5.01	0.3	CR	1	0
8	48/M	B-ALL MLL-AF4	Complex	No	5	Yes	118	79	0.86	4.1	CR	1	0
9	14/F	B-ALL TP53	Complex	Yes	15	Yes	88	63	1.91	1	CR	1	0
10	46/F	B-ALL	Complex	No	4	No	116	248	3.08	0.6	CR	1	0
11	20/M	B-ALL	Untested	No	5	Yes	90	74	1.52	2	CR	1	0
12	40/M	B-ALL	Complex	Yes	9	Yes	121	109	4.85	2	CR	1	0
13	22/F	B-ALL	Normal	No	9	Yes	130	127	2.77	2.79	CR	1	0
14	29/F	B-ALL RNUX1, IKZF1	Complex	Yes	4	Yes	116	143	2.33	1	CR	1	0
15	31/F	B-ALL WT1, E2A-HLF	Normal	No	7	Yes	78	59	3.79	4	CR	2	0
16	66/M	B-ALL	Complex	No	4	No	115	279	3.5	1	CR	3	0
17	20/M	B-ALL WT1	Complex	Yes	11	Yes	99	55	0.82	2	CR	2	0

M, male; F, female; B-ALL, B-cell acute lymphocytic leukemia; HSCT, hematopoietic stem cell transplantation; CRS, cytokine release syndrome; ICANS, immune effector cell-associated neurotoxicity syndrome; CR, complete remission.

^*^Acute lymphoblastic transformation of chronic lymphocytic leukemia.

### Efficacy and adverse events of CAR-T cells

The median follow-up time was 14.97 months (4.1–31.2 months). Twelve-month PFS and OS rates were 58.8% ( ± 11.9%) and 64.7% ( ± 11.6%), respectively. All patients had CRS. The incidence of grade 1/2 CRS was 88% (15/17), the incidence of grade 3 CRS was 12% (2/17), and no patients suffered neurotoxicity. CRS symptoms disappeared after symptomatic treatment.

**Figure 1:**
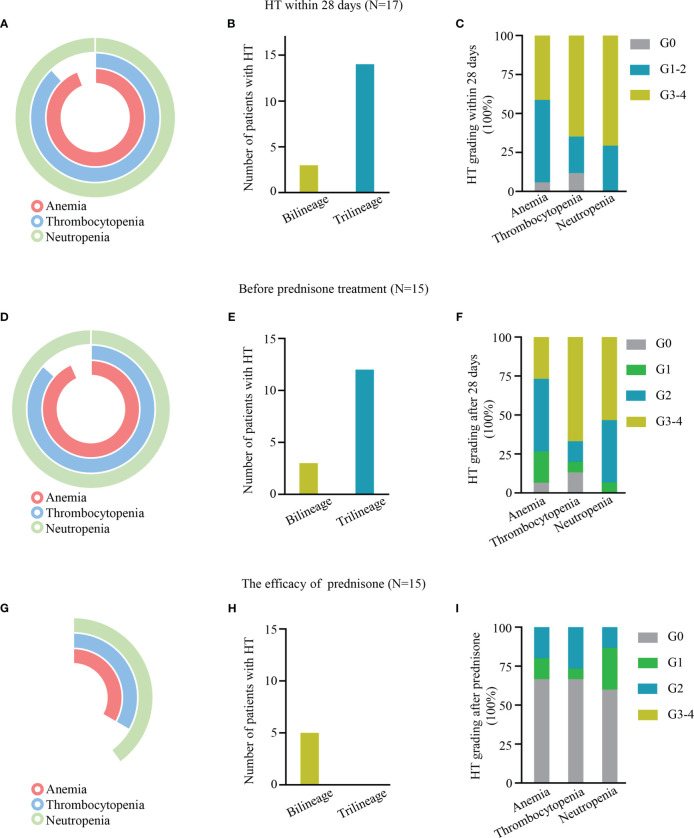
**(A–C, E–G, H–I)** Hematologic toxicity (HT) within 28 days, after 28 days, and after prednisone treatment, respectively, after chimeric antigen receptor T (CAR-T) cell treatment. **(A, D, G)** Pie charts depicting the incidence of anemia, thrombocytopenia, and neutropenia. **(B, E, H)** The number of HT in bilinear and trilineage response cytopenia. **(C, F, I)** The incidence of different levels of HT.

Within 28 days after CAR-T cell infusion, 96.12% (16/17) of patients had anemia, 88.24% (15/17) had thrombocytopenia, and 100% (17/17) had neutropenia (**Figure 1A**). The proportion of patients with trilineage cytopenia was 82.35% (**Figure 1B**). A majority of these events were grade 3–4 cytopenia, including 41.18% (7/17) of grade 3–4 anemia, 64.71% (11/17) of grade 3– thrombocytopenia, and 70.59% (12/17) of grade 3–4 neutropenia (**Figure 1C**).

Of the patients, 52.94% (9/17), 70.59% (12/17), and 82.35% (14/17) received hematopoietic treatment of blood transfusion, G-CSF, and drugs, such as erythropoietin and thrombopoietin receptor agonists, respectively. However, only 11.76% (2/17) of patients (P1 and P2) had complete hematologic recovery at D28; 88.24% (15/17) of patients still had varying degrees of cytopenia, defined as experiencing PHT; 80% (12/15) of patients had trilineage cytopenia, and 20% (3/15) of patients had bilineage cytopenia (**Figures 1D, E**). A total of 12 patients had grade 3/4 cytopenia, 26.67% (4/15) had grade 3/4 anemia, 66.67% (10/15) had grade 3/4 thrombocytopenia, and 53.33% (8/15) had grade 3/4 neutropenia (**Figure 1F**).

### Low dose of prednisolone is effective for PHT

The median duration between CAR-T cell therapy and prednisone was 30 days (range 26–42). The median response time was 21 days (7–40 days), and all patients’ blood count returned to safe levels (above G2) after prednisone treatment. Their hemoglobin count returned to standard levels in 66.67% (10/15) of patients, platelet in 66.67% (10/15), and neutrophil in 60% (9/15). Only 33.33% (5/15) of patients had bilineage cytopenia (**Figures 1G–I**).

### Low dose of prednisone therapy was effective in patients with repeated PHT

**Figure 2:**
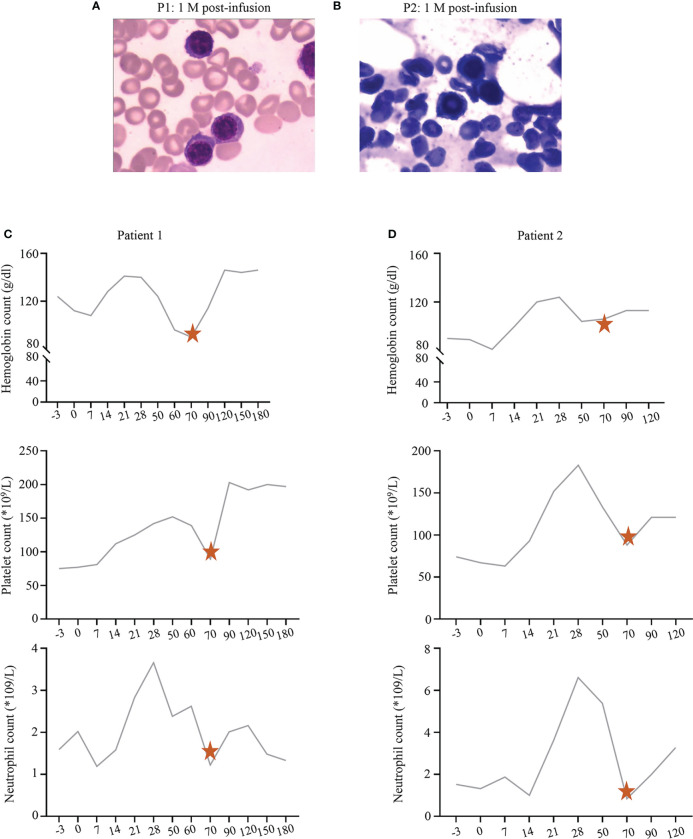
**(A, B)** The bone marrow status of P1 and P2 at 1 month post-infusion, respectively. **(C, D)** The recovery trend of hemoglobin, platelet, and neutrophil count in P1 and P2, respectively. The pentagrams represent the timing of prednisone administration.

The blood count of P1 and P2 had returned to normal levels after symptomatic treatment at D28. However, they developed unexplained cytopenia again around D70. They all maintained CR (**Figures 2A, B**). They were treated with prednisone, and their blood count returned to normal after 2 weeks of administration (**Figures 2C, D**).

**Figure 3:**
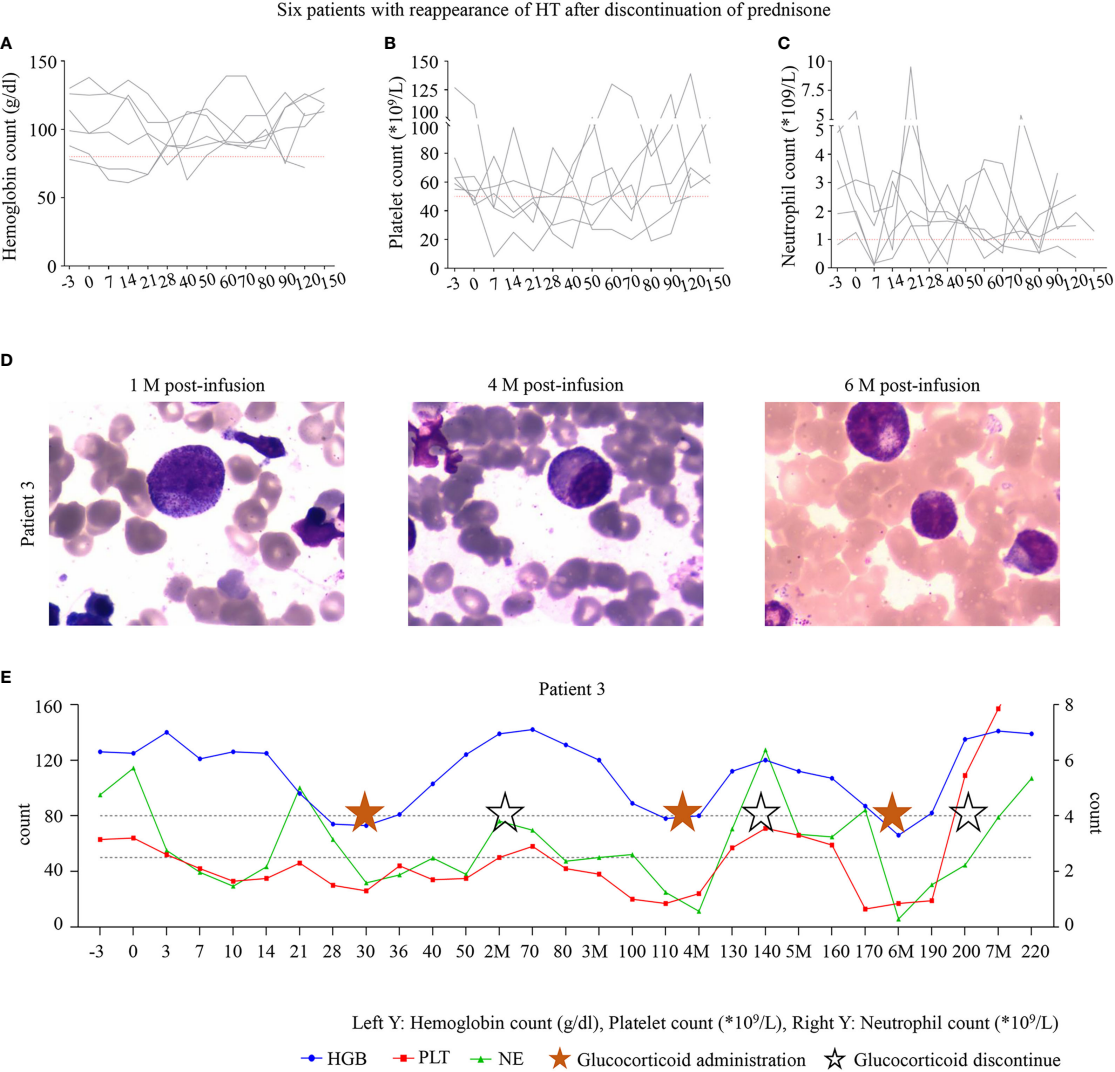
**(A–C)** The six patients with hematologic toxicity (HT) recurrence after discontinuation of prednisone. **(D)** The bone marrow of P3 at 1, 4, and 6 months after infusion. **(E)** The trend of blood cell count recovery in P3. P3 was administered with oral prednisone on D30 after infusion, and treatment was discontinued after hematologic recovery after 35 days of the administration. However, HT recurred after discontinuation. All three lineages were reduced to levels below grade 3 at month 4; prednisone was administered, and the patient recovered after 20 days. Trilineage reduction reappeared in month 6 after infusion and was alleviated again after prednisone administration. P3 is still undergoing follow-up. The time points marked with pentagrams are the duration of oral prednisone, and the hollow pentagrams represent the discontinuation of the drug.

Most interestingly, during the follow-up, we found that 40% (6/15) of patients developed HT again after stopping prednisone therapy and were effectively treated with prednisone therapy again (**Figures 3A–C**). P3 experienced grade 1 CRS, which disappeared soon after symptomatic management. During follow-up, P3 was in sustained remission (**Figure 3D**) but had grade 3/4 cytopenia. Therefore, he was started on oral prednisone on D30. His blood cell count recovered after treatment with prednisone, decreased again with discontinuation, and recovered again after administration. His blood cells fluctuated with the administration and withdrawal of prednisone (**Figure 3E**).

### Prednisolone is safe for PHT

No patients developed osteoporosis or gastrointestinal ulcers; 64.71% (11/17) of patients had hyperglycemia, and 29.41% (5/17) had hypertension, which recovered owing to oral hypoglycemic agents and antihypertensive drugs. Furthermore, after stopping prednisone treatment, the patients’ blood glucose and blood pressure were not abnormal. No patient developed infections during treatment with oral prednisone.

The effect of glucocorticoids on CAR-T cell expansion and efficacy is controversial. In our study, the peak of CAR-T cell expansion in most patients was between 7 and 14 days. We found CAR-T cells detectable in 35.29% (6/17) of patients before and after prednisone treatment and remained detectable in one patient even after 305 days (**Supplementary Material**). Therefore, low-dose steroids may have little effect on CAR-T cells.

## Discussion

The mechanism of HT is poorly understood, and the current treatment is mainly symptomatic such as transfusion of blood cells (16). We enrolled 17 patients who developed PHT after CAR-T cell therapy. Low-dose prednisone therapy promoted late hematologic recovery in these patients. More interesting was that cytopenia developed again after stopping the therapy and recovered after steroid therapy. Therefore, low-dose prednisone may be an optional treatment option for PHT secondary to CAR-T cell therapy.

HT after CAR-T cell treatment is typical. Early hematotoxicity may be associated with different treatment options, higher tumor burden, lymphoid depletion regimens before CAR-T cell infusion, and immune and hematopoietic system destruction by CAR-T cells (9, 17–20). HT will recover after the patients achieve symptomatic treatment, such as blood cell transfusion, G-CSF, and prevention or control of infection. However, more than half of the patients experienced PHT in our study, which was consistent with previous studies (17, 21). PHT will increase the risk of infection and bleeding in patients. Nevertheless, its mechanisms still need to be further studied.

More importantly, few studies have focused on the treatment of PHT. HSCT, immunosuppressant sirolimus, and TPO receptor agonists may benefit patients with PHT (22–26). Previous studies have shown that glucocorticoids could inhibit the immune responses of T cells and B cells, reduce the production of autoantibody, alleviate antigen–antibody response, and stimulate bone marrow hematopoiesis (27). We hypothesize that glucocorticoids may promote hematologic recovery in patients with PHT. We analyzed 17 B-ALL patients with PHT secondary to CAR-T therapy to evaluate the efficacy and safety of low-dose prednisone.

We included patients with persistent cytopenia who did not respond to traditional treatments, such as growth factors and blood-promoting drugs. Excitingly, after low oral doses of prednisone, all patients’ blood count recovered to safe levels (100%), with the complete recovery rate ranging from 60% to 66.67%. Interestingly, after stopping the drug, the blood cells decreased again in six patients. Their blood count recovered after continuing oral prednisone. In addition, a proportion of the patients had a biphasic pattern of HT. This pattern has been observed in previous studies (11). We found that low oral doses of prednisone were effective in patients with both sustained and bidirectional reductions.

Furthermore, during follow-up, OS and PFS were similar to those of the previous reports (28), and low-dose prednisone appeared not to affect CAR-T efficacy. We found that CAR-T cells could still be detectable after prednisone administration in six patients. This is consistent with studies that CRS glucocorticoid treatment does not affect the CAR-T cells’ efficacy and proliferation (29), even under high glucocorticoid doses (30). However, our study is a single-center study with a small number of cases and a short follow-up period, so it needs to be confirmed further.

Finally, we evaluated the safety of low-dose prednisone. We found that both hypertension and hyperglycemia were reversible and improved with symptomatic treatment. Small doses of prednisone should be safe for patients with PHT.

Our results show that prednisone promotes hematologic recovery in patients with PHT. Although we do not have sufficient evidence to demonstrate the mechanism of prednisone treatment, we speculate that PHT may be associated with abnormal immune activation. Excluding the effect of other secondary diseases on PHT and monitoring immune cell subsets and immune response factors may help to understand further the mechanisms by which prednisone therapy for PHT is effective.

In conclusion, our data suggest that low-dose prednisone may improve hematologic recovery in patients with PHT after CAR-T infusion and does not sacrifice the efficacy of CAR-T cells, and the side effects are manageable. However, validation in many cases and longer follow-up are still needed.

## Data availability statement

The original contributions presented in the study are included in the article/**Supplementary Material**. Further inquiries can be directed to the corresponding authors.

## Ethics statement

The studies involving human participants were reviewed and approved by the Institutional Review Board of Tianjin First Central Hospital. Written informed consent to participate in this study was provided by the participants’ legal guardian/next of kin.

## Author contributions

JW and MZ wrote the manuscript draft. MFZ and WL designed the study and managed the patients. HL, XX, XB, YP, JM, QL and TY contributed to patient management. MFZ and WL were responsible for clinical trial recruitment. JW and RG collected the data, and JW and MZ analyzed the data. MFZ and WL revised the manuscript. All authors contributed to the article and approved the submitted version.
