# Supplementary material for: Low-dose administration of prednisone has a good effect on the treatment of prolonged hematologic toxicity post-CD19 CAR-T cell therapy
Source: Front Immunol. 2023 Mar 14;14:1139559. doi: 10.3389/fimmu.2023.1139559 (PMC10043253; doi:10.3389/fimmu.2023.1139559)
Supplement: Supplementary file 1 [file DataSheet_1.docx]

**Supplementary data**


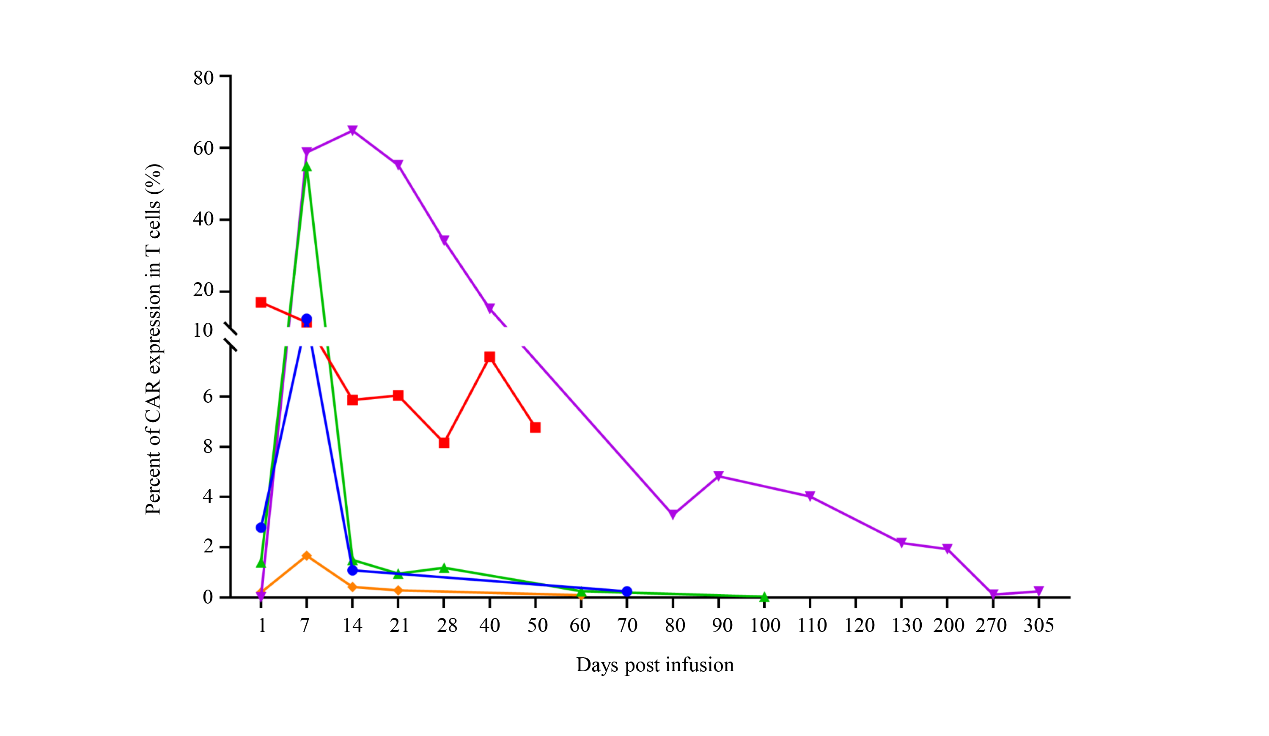
CAR-T cells were detectable in all six patients before and after prednisone treatment. All patients used flow cytometry except the last one who used qPCR (Table 4) to monitor CAR-T cells.

| Table: CAR-T cells expansion of patient 4 | | | | |
| --- | --- | --- | --- | --- |
|  | D7 | D14 | D28 | D50 |
| Patient 4 | 7.9*10^8^ | 6.4*10^8^ | 2.6*10^8^ | 3.2*10^8^ |
